# Integrative bioinformatics analysis identifies ROBO1 as a potential therapeutic target modified by miR-218 in hepatocellular carcinoma

**DOI:** 10.18632/oncotarget.18099

**Published:** 2017-05-23

**Authors:** Junqing Wang, Yunyun Zhou, Xiaochun Fei, Xunhua Chen, Rui Chen, Zhenggang Zhu, Yongjun Chen

**Affiliations:** ^1^ Department of Surgery, Ruijin Hospital, Shanghai Jiao Tong University School of Medicine, Shanghai 20025, People’s Republic of China; ^2^ Shanghai Institute of Digestive Surgery, Ruijin Hospital, Shanghai Jiao Tong University School of Medicine, Shanghai 20025, People’s Republic of China; ^3^ Shanghai Key Laboratory of Gastric Neoplasms, Ruijin Hospital, Shanghai Jiao Tong University School of Medicine, Shanghai 20025, People’s Republic of China; ^4^ Department of Data Science, University of Mississippi Medical Center, Jackson, MS 39216, USA; ^5^ Department of Pathology, Ruijin Hospital, Shanghai Jiao Tong University School of Medicine, Shanghai 20025, People’s Republic of China; ^6^ Department of Plastic and Reconstructive Surgery, Renji Hospital, Shanghai Jiao Tong University School of Medicine, Shanghai 200001, People’s Republic of China

**Keywords:** hepatocellular carcinoma, bioinformatics analysis, ROBO1, MiR-218

## Abstract

Patients diagnosed with advanced hepatocellular carcinoma (HCC) presented poor prognosis and short survival time. Althouth accumulating contribution of continuous research has gradually revealed complex tumorigenesis mechanism of HCC with numerous and jumbled biomarkers, those specific ones for HCC diagnose and therapeutic treatment are required illustration. Multiple genes over-expressed in HCC specimens with at least 1.5 fold change were cohorted, compared with the non-cancerous tissues through integrative bioinformatics analysis from Gene Expression Omnibus (GEO) datasets GSE14520 and GSE6764, including 445 and 45 cases of samples spearatly, along with intensive exploration on the Cancer Genome Altas (TCGA) dataset of liver cancer. Thirteen genes significantly highly expressed, overlapping in the datasets above. The Database for Annotation Visualization and Integrated Discovery (DAVID) program was utilized for functional pathway enrichment analysis. Protein-protein Interaction (PPI) analysis was conducted through the Search Tool for the Retrieval of Interacting Genes (STRING) database. ROBO1 was highlighted as one of the most probable molecules among the 13 candidates participating in cancer process. Cancer Cell Line Encycolopedia (CCLE) database was utilized exploring ROBO1 expression in cell lines. Immunochemistry analysis and qRT-PCR assay were performed in our medical center, which indicates significant over-expression status in either HCC tumor specimens and 3 HCC cell lines. Furtherly, we recognized that miR-218, a tumor suppressor, might be an upstream regulator for ROBO1 directly binding to the mRNA 3’UTR and potentially modifying the expression and function of ROBO1. Herein, we conclude that ROBO1 is a mighty therapeutic targets modified by miR-218 in HCC deserving further investigation.

## INTRODUCTION

Hepatocellular carcinoma (HCC) ranks the fifth most common malignancy globally and the second cause of mortality among the patients suffering from malignant tumors [1, 2]. Accumulating evidence and numerous mighty biomarkers involved in HCC tumorigenesis have been discovered, from transcriptional and post-transcriptional regulation to methylation and phosphorylation process. However, findings correlated with HCC have not been integrated perfectly, and specific targets for HCC diagnose and therapeutic treatments are required illustration intensively.

Gene Expression Omnibus (GEO) database and the Cancer Genome Altas (TCGA) database provide a possibility for bioinformatic mining of gene expression profile involved with cancerous diseases. By integratively analysis of datasets from either GEO or TCGA database, we successfully corhorted a set of differential expression genes (DEGs), which potentially participate in tumorigenesis and progress of HCC.

In this study, 13 genes showed significant over-expression status in HCC tissues through the bioinformatics analysis of two GEO datasets, GSE14520 and GSE14323 datesets, and the TCGA liver cancer datasets [3, 4]. According to functional pathway analysis by using the Database for Annotation Visualization and Integrated Discovery (DAVID) and Protein-protein Interaction (PPI) analysis, Roundabout Guidance Receptor 1 (ROBO1) was noticed as one out of the thirteen candidate genes, which might be the most probable molecule involving in HCC cell proliferation and mobility.

As knowledged, ROBO1, also named as Protein-SAX3, is the receptor of Slit Guidance Ligand 1 and 2 (SLIT1, SLIT2), which associate with cell migration either through GTPase activity process or molecular guidance cues response [5, 6]. And several reports have demonstrated ROBO1 as a promotor of cell proliferation in human maligancies, such as bladder cancer and glioblastoma [7, 8]. While, evidence for ROBO1 function in HCC is not sufficient so far, and is necessary to be further illustrated.

We checked Cancer Cell Line Encycolpedia (CCLE) database to explore ROBO1 expression level in mutiple HCC cell lines. Also, we carried out qRT-PCR assay and immunochemistry analysis in 23 HCC paired samples and cell line (QSG-7701, Hep3B and HePG2) in our medical center, to determine the ROBO1 expression characteristic. Along with the observation of datasets, ROBO1 showed aberrantly highly expression in both HCC specimens and cell lines. Furtherly, we predicited miR-218, one of the validated post-transcriptional tumor suppressors in a serie of tumors including HCC, as a potential regulator of ROBO1. Dual-luciferase report assay was carried out in Hep3B cells, and miR-218 indicated directly interaction with the 3’ untranslated region of ROBO1 mRNA. This illustrates that miR-218 suppresses ROBO1 expression in HCC cells. All above combining with integrative bioinformative analysis suggest ROBO1 as a potential therapeutic target modified by miR-218 in HCC.

## RESULTS

### Selection of DEGs over-expressed in HCC

**Figure 1:**
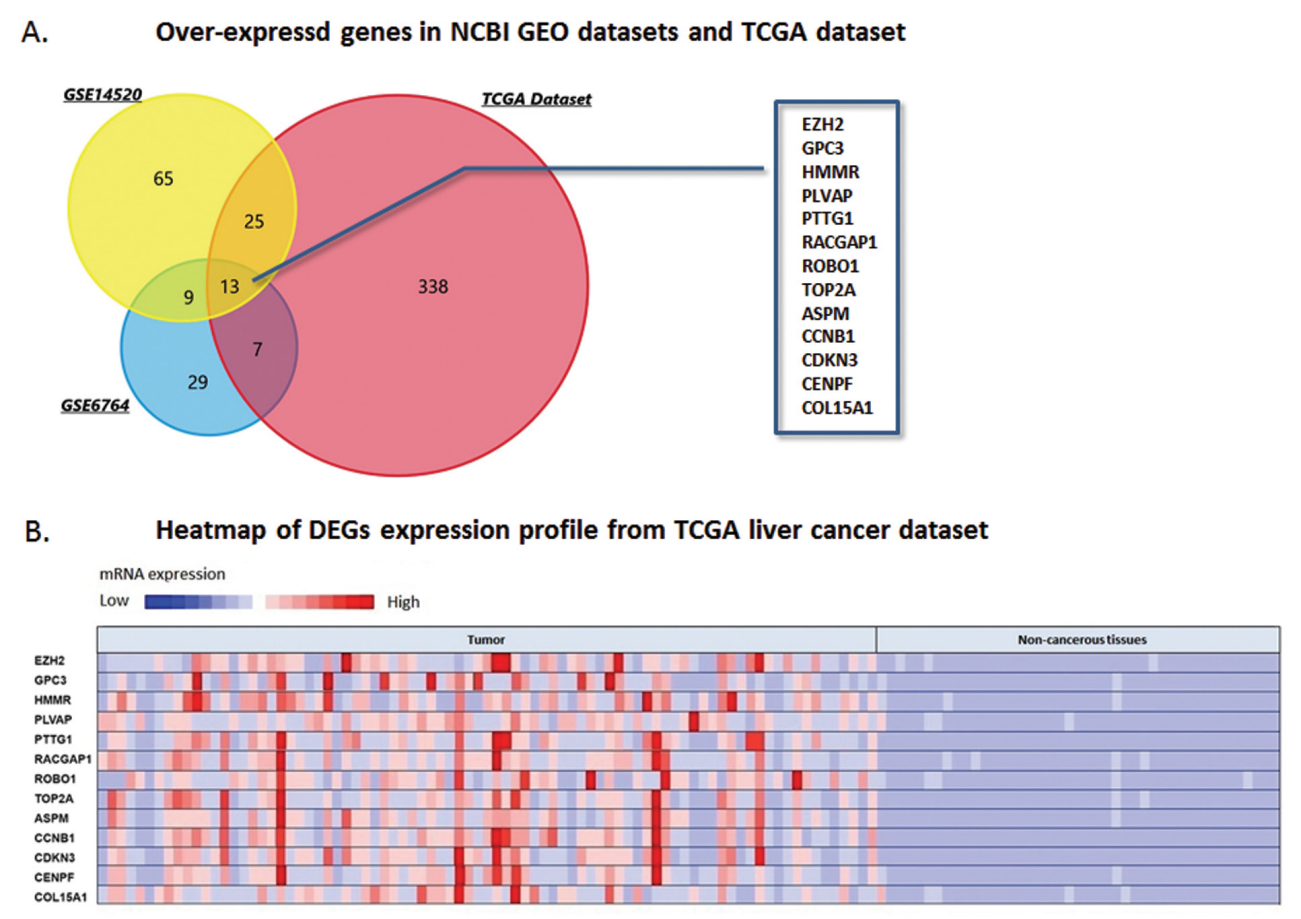
DEGs over-expressed in NCBI GEO datasets and TCGA database **(A)** Venn chart of the over-expressing genes in GSE14520, GSE6764 and TCGA live cancer datasets, which overlapped according to the analysis of FunRich software. Thirteen DEGs over-expressed in the three datasets were selected, including ROBO1, EZH2, GPC3, HMMR, PLVAP, PTTG1, RACGAP1, TOP2A, ASPM, CCNB1, CDKN3, CENPF, and COL15A1. **(B)** Heatmap generated from TCGA liver cancer dataset. Expressions of the thirteen DEGs above were presented by hot spots with gradient from red as high expression to blue as low expression. Expression of the DEGs shows significantly high level in HCC tumor tissues than that of non-cancerous tissues.

According to the criteria for selecting DEGs, 608 genes present over-expression status in HCC tissues than that of non-cancerous samples were set. Among these genes, we excluded 99 candidates which could not be mapped. And the rest 509 DEGs were calculated through FunRich software. As Figure 1A shown, thirteen genes including ROBO1, EZH2, GPC3, HMMR, PLVAP, PTTG1, RACGAP1, TOP2A, ASPM, CCNB1, CDKN3, CENPF and COL15A1 were observed over-expressed and overlapped among three datasets of this study. Analysis of the expression fold-change of the DEGs showed extremely significant difference at mRNA levels between tumor and normal liver tissues, which was partly demonstrated in Figure 1B.

### Functional pathway of the DEGs and the selection of ROBO1

**Figure 2:**
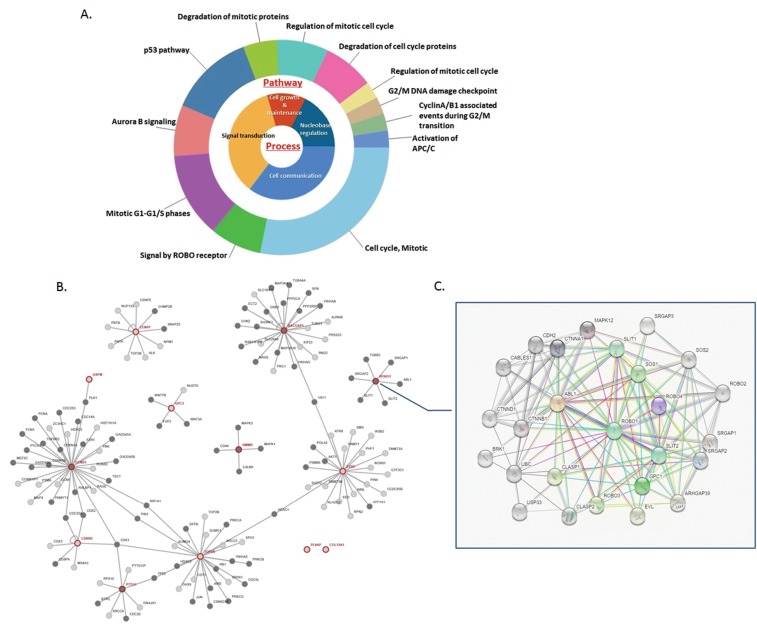
Molecular functional pathways and process of DEGs and selection of ROBO1 **(A)** Pie chart for illustration of DEGs molecular pathways and process analyzed by DAVID online tools. Most of the molecular pathways are associated with mitotic phase transition and cell cycle, along with the main process of cell growth and maintenance, signaling transduction, cell communication and nucleobase regulation. **(B)** On line tool of STRING database analysis of the PPI network for the DEGs. **(C)** Amplification of the network for PPI associated with ROBO1, which demonstrates a complex network between ROBO1 and proteins pivotal in tumorigenesis and process.

Table 1 shows the result of the enrichment analysis for DEGs in this study. These thirteen up-regulated genes were enriched, and we noticed significant enrichment of processes correlated with cancer genesis and progress, including regulation of cellular protein metabolic process (GO:0032268), apoptotic process (GO:0006915), regulation of intracellular signal transduction (GO:1902531), cell cycle (GO:0007049), cell cycle phase transition (GO:0044770), pathways in cancer (Hsa05200), signaling pathways regulating pluripotency of stem cells (Hsa04550), central carbon metabolism in cancer (Hsa05230), transcriptional mis-regulation in cancer (Hsa02520) and mTOR signaling pathway (Hsa04150). Occasionally, we recognized that ROBO1, one of the 13 candidate DEGs, was involved in cancer pathway. As demonstrated, the molecular pathways and process were calculated associated with the 13 DEGs, and PPI network was generated from STRING online tool and Cytoscape software (Figure 2A, 2B). The DEGs showed distinguished networks and interaction. ROBO1, as we expected, presented a complex protein to protein interaction described in detail as Figure 2C shown.

**Table 1:** Presentation of part of the GO and KEGG pathway enrichment analysis for the up-regulated DEGs associated with cancer genesis and progress

Category	ID	Term	*P*-value
BP	GO:0000278	Mitotic cell cycle	1.5E-36
BP	GO:0007049	Cell cycle	8.4E-35
BP	GO:0051246	Regulation of protein metabolic process	1.7E-24
BP	GO:0032268	Regulation of cellular protein metabolic process	1.1E-24
BP	GO:0044770	Cell cycle phase transition	1.0E-22
BP	GO:0031400	Negative regulation of protein modification process	6.1E-19
BP	GO:0006915	Apoptotic process	3.2E-13
BP	GO:0012501	Programmed cell death	1.4E-13
BP	GO:1902531	Regulation of intracellular signal transduction	7.2E-13
BP	GO:0006974	Cellular response to DNA damage stimulus	2.0E-13
BP	GO:0008283	Cell proliferation	7.4E-13
BP	GO:0000165	MAPK cascade	4.4E-11
KEGG pathway	Hsa05200	Pathways in cancer	2.2E-9
KEGG pathway	Hsa05205	Proteoglycans in cancer	1.1E-8
KEGG pathway	Hsa04150	mTOR signaling pathway	1.4E-4
KEGG pathway	Hsa04550	Signaling pathways regulating pluripotency of stem cells	3.2E-3
KEGG pathway	Hsa02520	Transcriptional misregulation in cancer	2.2E-3
KEGG pathway	Hsa05230	Central carbon metabolism in cancer	1.2E-2

### ROBO1 expresses aberrantly high level in HCC tissues and cell lines

**Figure 3:**
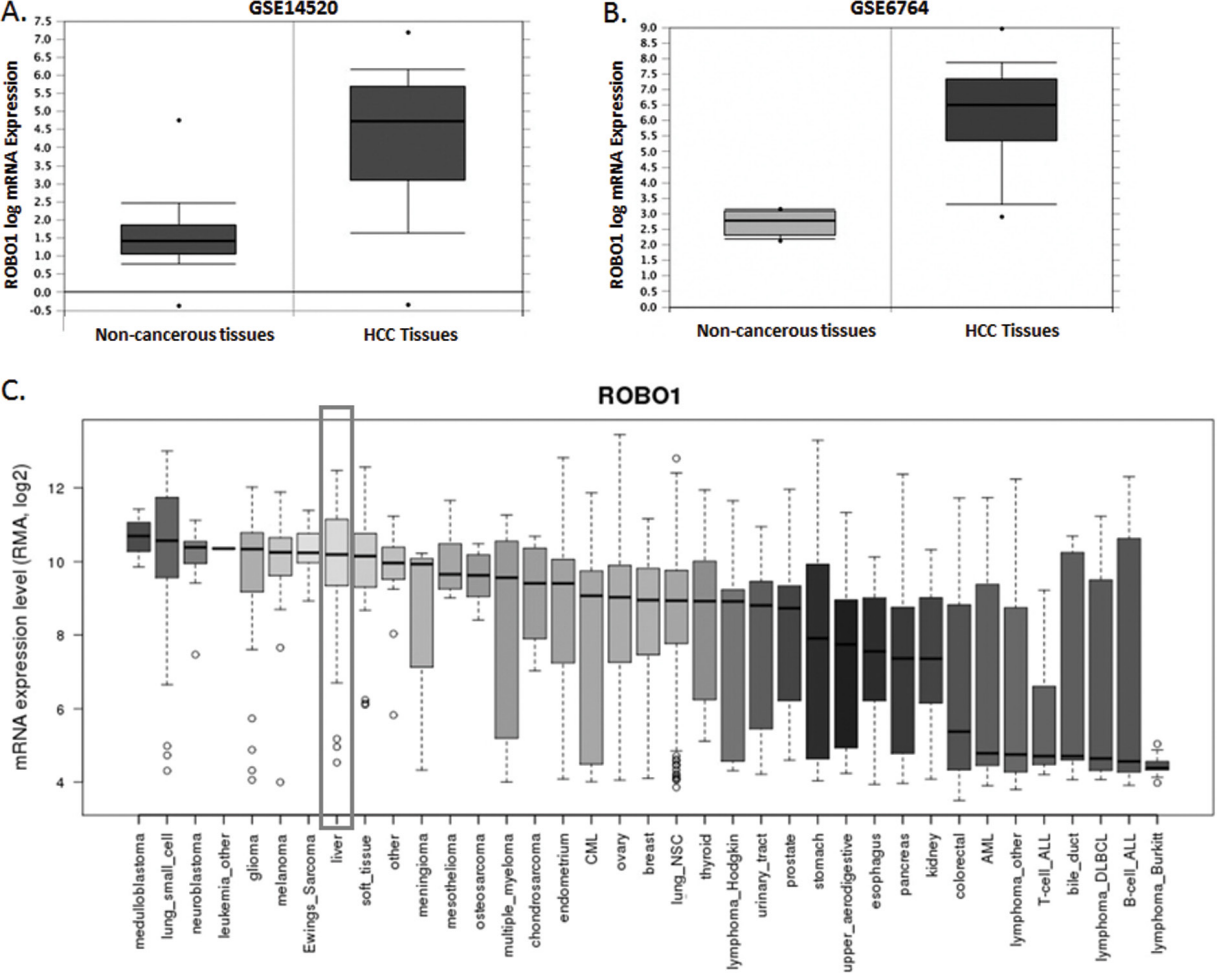
ROBO1 expression profile in NCBI GEO datasets and differential human malignancies **(A)** Comparison of ROBO1 mRNA level between non-cancerous tissues and HCC tumor in GSE14520 datasets. ROBO1 presented a significant higher expression in HCC tissues than the non-cancerous tissues (*P*=1.17E-65). **(B)** Comparison of ROBO1 mRNA level between non-cancerous tissues and HCC tumor in GSE6764 datasets. ROBO1 presented a significant higher expression in HCC tissues than the non-cancerous tissues (*P*=1.53E-15). **(C)** ROBO1 expression in differential human malignancies from CCLE database. HCC tissues expressed relatively higher ROBO1 compared with most of the other human cancers in a reliable confidence interval.

According to the primary explore from NCBI GEO database (GSE14520 and GSE6764), ROBO1 was expressed extremely higher than that of in normal liver tissues (*P*=1.17E-65 for GSE14520 and *P*=1.53E-15 for GSE6764) (Figure 3A, 3B). We then searched the online database of CCLE to further exam the expression status of ROBO1.

**Figure 4:**
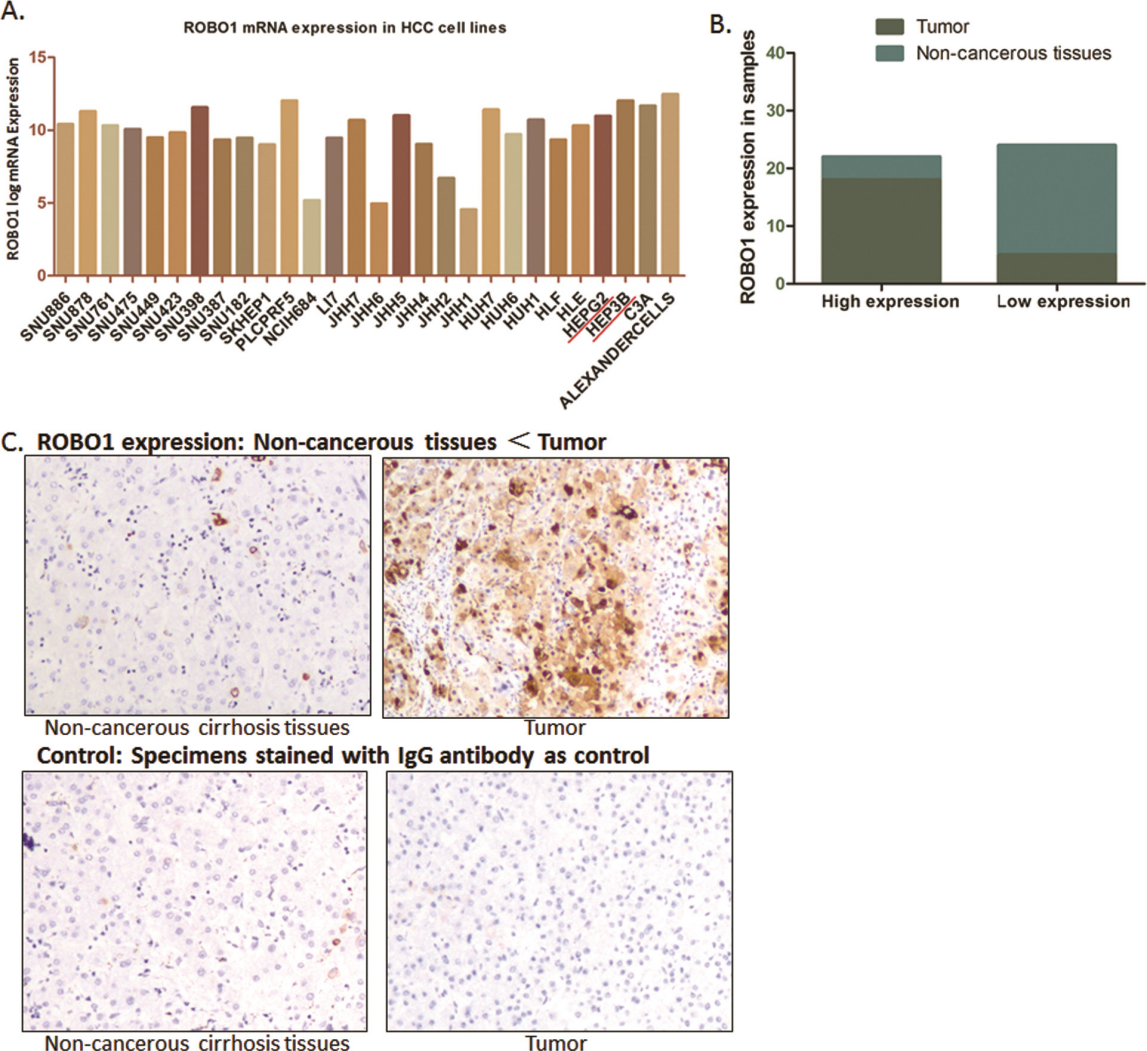
ROBO1 expression in HCC cell lines and real patients’ specimens **(A)** ROBO1 expression in different HCC cell lines from CCLE database. As demonstrated, ROBO1 mRNA level is commonly high in various HCC cell lines. **(B)** Expression of ROBO1 was up-regulated in most of the tumor tissues (18/23), and was expressed at a lower level in most of the adjacent non-cancerous tissues (4/23). The expression of ROBO1 in tumor tissues was frequently and significantly higher than adjacent non-cancerous tissues (*P*<0.05). **(C)** Representative graph immunohistochemistry analysis carried out on tissue microarray (200×).

In Figure 3C, we presents a comprehensive profile of ROBO1 inclining to be amplified in distinguish human malignancies. Tumor primary from liver indicates a reliable higher expression of ROBO1 among the 37 tumors. We further detected the expression variation between HCC cell lines. As Figure 4A demonstrated, ROBO1 level in differential HCC cell lines is commonly high.

Thus, we went forward to validate the ROBO1 expression characteristic in 23 paired HCC samples and 3 HCC cell lines. Seventeen paired specimens of HCC tumor tissues and adjacent non-cancerous tissues of HCC patients from our medical center were prepared and detected through IHC. According to the expression intensity of ROBO1, these cases were separated by ROBO1 into low and high expression group. Among the tumor specimens, 78.26% (18/23) of the cases presented high level ROBO1, and only 21.74% (4/17) cases showed relatively lower RGS3 expression. For adjacent non-cancerous tissues, high ROBO1 was distinguished only in a small portion of the cases (17.40%, 4/17) out of the 17 specimens, with an 82.60% (19/23) portion presents lower level of ROBO1 (Figure 4B, 4C). Although cases recruited in this study did not provide large samples, it suggests that ROBO1 expresses frequently higher in HCC tissues than the normal tissues.

In HCC cell lines (SMMC-7721, HePG2 and HeP3B), we also observed that the mRNA level of ROBO1 is significantly differentially expressed in three HCC cell lines compared with QSG-7701 cells (P<0.05) (Figure 4D). Hep3B, which presents a high level of ROBO1 expression from CCLE database, was recognized expressing highest ROBO1 mRNA among the three HCC cell lines. The high expression characteristic of RGS3 in GC cells is consistent with the observation through IHC of tumor tissues.

### MiR-218 directly binds to the 3’ UTR of ROBO1 mRNA

**Figure 5:**
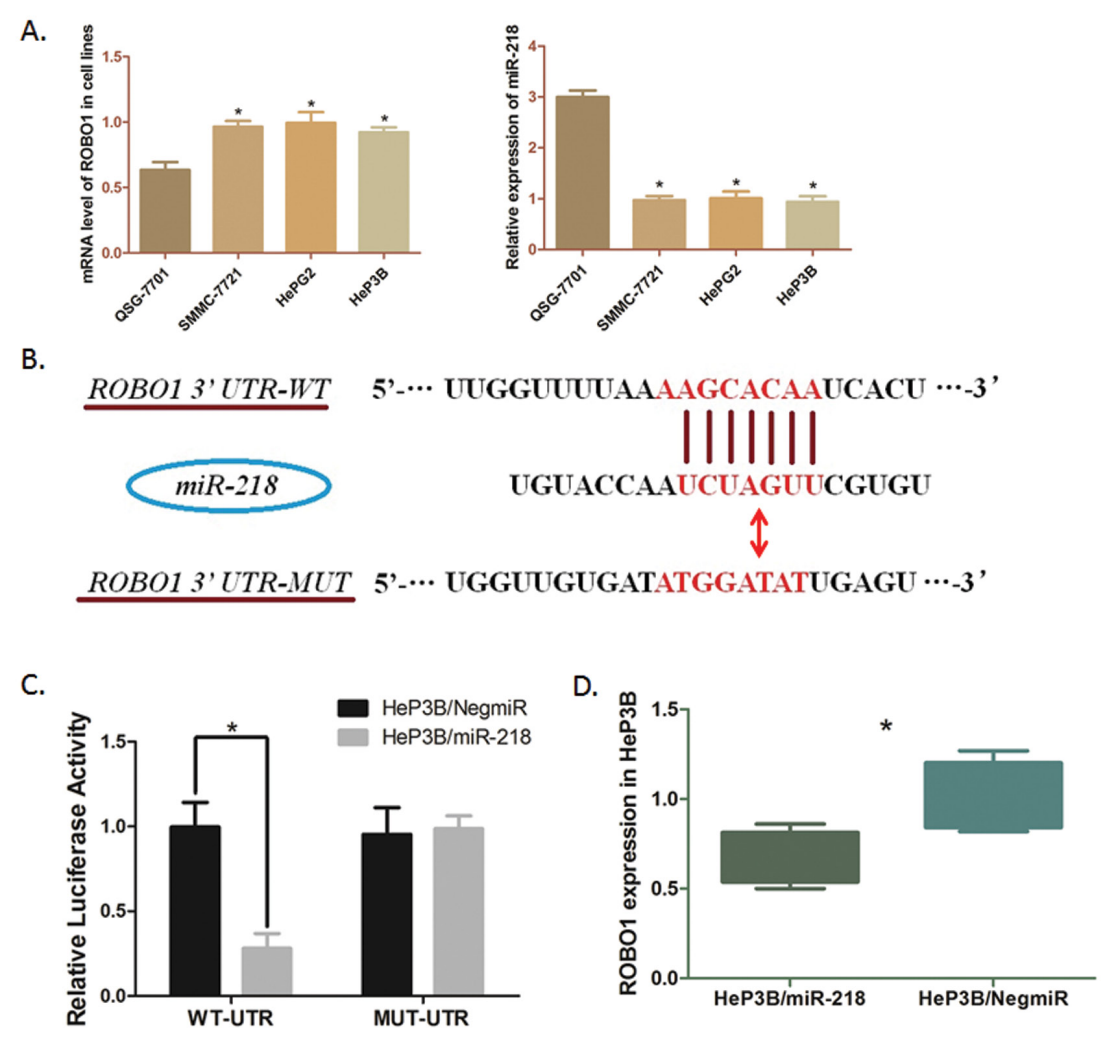
Modulation of ROBO1in HeP3B cells directly regulated by miR-218 **(A)** Transcription level of ROBO1 and miR-218 in cell lines were detected by qRT-PCR. The mRNA level of ROBO1 is significantly higher in HCC cells than that in QSG-7701 cells (*P<0.05), and the level of miR-218 is obviously down-regulated in HCC cells compared with QSG-7701 (*P<0.05). Results above are means of three independent experiments ± SD. **(B)** Scheme for the predicted miR-218 binding site in the wild type ROBO1 mRNA 3’UTR (3’UTR-WT), and in the mutant construct (3’UTR-MUT). **(C)** The direct interaction between miR-218 and ROBO1 was detected by Dual-luciferase reporter assay. Over-expression of miR-218 in HeP3B cells (HeP3B/miR-218) significantly decreased the luciferase signal of ROBO1/pMIR/WT compared with the negative control (HeP3B/NigmiR) (*P<0.05). And mutation of the putative miR-218-binding site abolished this suppressive effect. **(D)** QRT-PCR was undertaken to detect the effect of miR-218 on ROBO1 mRNA level in HeP3B cells. ROBO1 expression was significantly suppressed by over-expressing miR-218 in HeP3B cells. The results are means of three independent experiments ± SD. (*P<0.05).

We applied bioinformatics analysis and online prediction software utilizing miRDB and TargetScan, to predict potential upstream binding microRNA of ROBO1. MiR-218, a validated suppressor of HCC, presented a mighty post-transcriptional regulator of ROBO1 (Figure 5A). QRT-PCR was conducted, which indicated a relatively lower expression of miR-218 in HCC cell lines than that of in QSG-7701 cells (Figure 4D). Validation of the direct interaction between ROBO1 mRNA and miR-218 was carried out by Dual-luciferase reporter assay. The luciferase reporter vectors containing 200 bp 3’-UTR sequence of ROBO1 (WT-UTR) and the corresponding control luciferase vectors containing mutated miR-218 target site (MUT-UTR) were constructed. As Figure 5B shown, over-expression of miR-218 in Hep3B cells (Hep3B/miR-218) negative control (Hep3B/NigmiR). And, this suppressive effect induced by miR-218 was significantly abolished in Hep3B cells with the putative binding site of miR-218 mutated. Moreover, the mRNA level of ROBO1 was significantly decreased in Hep3B /miR-218 cells (Figure 5C). Taken together of these results, ROBO1 is a direct target in GC cells post-transcriptionally down-regulated by miR-218.

## DISCUSSION

HCC is a highly aggressive neoplasm of humanity, which causes poor outcome in patients with advanced stages [9]. Quite a lot of patients suffering HCC were diagnosed at relatively later status, no longer applicable for radical resection, and weak response to chemotherapy associated with multi-drug resistance leaves us rigorous challenge in HCC therapeutic treatment [10]. In preclinical and clinical research, molecule-targeted therapy has been focused on for a remarkable therapeutic strategy [11, 12]. However, sorafenib seems to be the only effective molecule-targeted medicine approved by clinical practice so far [13, 14]. And researchers are continuously seeking for new biomarkers or targets for HCC prevention, diagnose and treatment.

Numerous molecules, including non-coding RNAs, transcriptional and post-transcriptional products, have been revealed expressing aberrantly in HCC. These molecules connect and establish complex network in HCC genesis and progress. Intensive data mining of these genes’ information could be applied from NCBI GEO and TCGA databases, which provide possibility for integrative learning of the expression profiles and the relative functional network.

In our study, we downloaded datasets of HCC patients’ information and gene expression data from NCBI GEO database (GSE14520 and GSE6764) and TCGA database. The DEGs expressed significantly higher in HCC tissues than that of in normal live tissues were mapped and focused on in this study.

608 genes with significant over-expression characteristic in HCC tissues were selected from the three datasets above, according to the fold change of mRNA level compared with normal liver tissues. We used FunRich software to analysis these genes, and got a map of 13 DEGs overlapping in all datasets. GO and KEGG enrichment pathway was conducted. Combined with the results of enrichment, we noticed a set of pathways involve in tumorigenesis and process, including protein metabolic, cell apoptosis, intracellular signal transduction, cell cycle phase transition, stem cell pluripotent regulation, mTOR signaling pathway and pathway in cancer. Thus, we got a comprehensive view of the molecular pathway and function of these 13 candidates. As Figure 2A demonstrated, mitotic phase transition and cell cycle were involved as pivotal pathways enriched among the 13 genes, and the main process of these molecules are cell growth and maintenance, signaling transduction, cell communication and nucleobase regulation. And, ROBO1, a SLIT1 and SLIT2 receptor involves in cell migration, was noticed in our study. We predicted ROBO1 as one of the pivotal molecules correlative with HCC.

ROBO1 belongs to Roundabout protein family. So far, there have been four members found in this family, from ROBO1 to ROBO4. Distinguished from ROBO3 and ROBO4, which are expressed specifically in central nervous system and endothelial cells, ROBO1 is conserved and expressed in most of the tissues in human being [15–17].

The main function of ROBO1 is to interact with Slit Guidance Ligand (SLIT) as an axon guidance receptor. The Slit-ROBO1 interaction was firstly described that transducts signals modifying repulsive cues on axons and growth cones in neural development and regulates chemotaxis of T cells and monocytes [18, 19]. Accumulating evidence has validated that ROBO1 performs differential expression profile and functions in various human malignancies. In gastric cancer (GC), ROBO1 is frameshift mutated and causes a loss of expression in 29% of GC samples, which intimates ROBO1 as one of the potential tumor suppressor in GC [20]; In brain specific metastasis of breast cancer, there also presents a low expression of ROBO1, which associated with poor prognosis [21]. On the contrary, ROBO1 behaves as tumor promotor in some other maligancies like lung cancer, in which high expression of ROBO1 is suggested as an independent player promoting brain metastasis [22]. Additionally, ROBO1 effects on tongue carcinoma cells to enhance the ability of cell adhesion, invasion and migration by alerting the expression of matrix metalloproteinase and E-cadherin, which [23]. Function of ROBO1 in HCC is controversial. Some reports indicate that ROBO1 acts as a kind of HCC promotor through contributing for cancerous angiogenesis [24, 25]. Nevertheless, evidence for the exact role of ROBO1 in HCC and its regulation mechanism still demand exploration.

In this study, we firstly confirmed ROBO1 over-expression profile in HCC tissues by analyzing both NCBI GEO datasets and TCGA database. Then, CCLE database was utilized to further validate the expression characteristic of ROBO1 in differential tumor cell lines. As we observed, ROBO1 is highly expressed in most of the tumor cell lines, and in HCC tissues, the expression ROBO1 is extremely high within a reliable confidence interval. Within differential HCC cell lines CCLE database collected, ROBO1 demonstrates an overall over-expression as we compared. These results strongly suggest ROBO1 as a potential significant gene in HCC.

To further validate the findings we discovered through data mining, we used paired tumor tissues and three cell lines from our own medical center for research. Although the samples were limited, a significant over-expression of ROBO1 was observed by applying IHC assay in HCC samples compared with the paired non-cancerous tissues. Simultaneously, qRT-PCR assay demonstrated significant difference between 3 HCC cell lines and the control QSG-7701 cells.

MiR-218 is one of the microRNAs described as tumor suppressor in various malignancies. In lung cancer, down-regulation of miR-218 affects the Slug/ZEB2 signaling pathway and consequentially causes epithelial-mesenchymal transition and metastasis [26]. MiR-218 involves in inhibition of GC cell growth and invasion through targeting Angiopoietin-2 [27]. Additionally, in prostate cancer, miR-218 acts as a suppressor for tumor cell proliferation [28]. For HCC, miR-218 has been validated as a down-regulated genes associated with tumor process through targeting different downstream mRNAs, such as PTEN, E2F2 [29, 30]. However, the relative research is not sufficient. Occasionally, we used online prediction tools and found a potential binding site of ROBO1 mRNA 3’-UTR, which could interact with miR-218.

Since the opposite expression characteristic of miR-218 and ROBO1, we hypothesized a direct interaction between this two molecules. Thus, Dual-luciferase reporter assay was conducted. The luciferase signal was significantly decreased when miR-218 was elevated in Hep3B cells, which indicated a direct binding of miR-218 and ROBO1 3’-UTR. Herein, we illustrate ROBO1 as a direct downstream target of miR-218 in Hep3B cells. However, limited by the inadequate support of experiment either *in vivo* or *in vitro*, further research is required to find out more dependable evidence. Whatever, according to discovery in this study, it is clear that ROBO1 is a hopeful therapeutic target negatively modulated by miR-218 in a post-transcriptional way.

In summary, through integrative analysis of NCBI GEO datasets and TCGA database, we got a comprehensive image of the expression profile of DEGs. Combining with functional pathway enrichment analysis and PPI study, ROBO1 presents to be a mighty molecule highly expressed and functioning in HCC. *In vitro* experiment along with clinical specimen research validated the expression profile of ROBO1 in both HCC samples and cell lines. Moreover, miR-218, a predicted upstream regulator was proved to be the direct modulator in HCC cells affects ROBO1 expression. ROBO1 should be a mighty gene for therapeutic target in HCC post-transcriptionally regulated by miR-218.

## MATERIALS AND METHODS

### Gene expression profile data

The gene expression profile dataset GSE14520 and GSE6764 was downloaded from GEO database (https://www.ncbi.nlm.nih.gov/geo/). Platforms of GEO datasets are GPL3921 (Affymetrix HT Human Genome U133A Array) for GSE14520 dataset and GPL570 (Affymetrix Human Genome U133 Plus 2.0 Array) (Agilent Technologies, Santa Clara, CA, USA) for GSE6764. In GSE14520 dataset, there consists of 445 samples derived from HCC patients (225 tumor tissues and 220 non-cancerous tissues). As for GSE6764, 45 samples were included with 35 tumor tissues and 10 non-cancerous tissues. Another dataset of liver cancer gene expression profile was obtained from TCGA database by USCS Refseq Gene Array containing gene profile of 84 HCC tumor samples and 42 paired adjcent non-cancerpis samples (https://tcga-data.nci.nih.gov/tcga/).

### DEGs identification

Data downloaded was preprocessed including background correction and transformation from probe level to gene symbol through R language conducted by two professional bioinformatics analysts, followed by normalization. DEGs between HCC samples and non-cancerous tissues were selected and corhorted basing on a t-test of linear models for microarray analysis package in R (Version 3.3, http://www.bioconductor.org) [31]. Fold-change (FC) of gene expression was calculated with a threshold criteria of log2FC≥1.5 and *P* value<0.01 for DEG selection. Funrich Software (Version 3.0, http://funrich.org/index.html) was utilized to analyze the DEGs overlapping characteristic among the three datasets. Thirteen candidate genes over-expressing in HCC samples were set as cohort.

The online database of Cancer Cell Line Encyclopedia (CCLE: https://portals.broadinstitute.org) was applied to determine the expression level of candidate gene among differential HCC cell lines.

### Functional network establishment of DEGs candidates

GO and KEGG pathway enrichment analysis were performed to investigate the functiions and processes of the three candidate DEGs, by applying online tools of the Database for Annotation Visualization and Integrated Discovery (Version 6.7, https://david.ncifcrf.gov/), a reliable program integrating and demonstrating the functional annotations of either genes or proteins. The cut-off value for significant function and pathway screening was set as P<0.01. The Search Tool for the Retrieval of Interacting Genes (STRING) database (Version 10.0, http://string-db.org) was recruited to predict the potential interaction between candidate genes at protein level. Cytoscape software (Versionn 3.4.0, http://www.cytoscape.org/) was used to construct the network of PPI. ROBO1 was noticed as a mighty candidate pivotal in HCC process.

### Cell culture and surgical specimens

The normal human hepatic cell line QSG-7701 and three HCC cell lines (SMMC-7721, HePG2 and HeP3B) were purchased from Shanghai Institutes for Biological Sciences, Chinese Academy of Science (Shanghai, China). Hep3B cells over-expressing miR-218 and the control one were constructed for Dual-luciferase report assay following the methods and protocol as we previously described [32, 33]. All cells were cultured in RPMI 1640 supplemented with 10% heat-inactivated fetal bovine serum (FBS), 100 ug/ml streptomycin and 100U/ml Penicillin in a humidified cell incubator at 37°C with an atmosphere of 5% CO_2_.

Twenty-three pairs of HCC cancer specimens along with the adjacent non-cancerous tissues were collected from HCC patients performed partial hepatectomy without preoperative therapy at the Department of Surgery, Ruijin Hospital, Shanghai Jiao Tong University School of Medicine within 2015.

### Immunohistochemistry analysis and qRT-PCR assay

Antibodies against ROBO1 and GAPDH (Abcam, USA) were purchased, and horseradish peroxidase-conjugated secondary antibody (Abcam, USA) was prepared. Immunohistochemistry analysis was carried out. Antibody against ROBO1 was used following the manufactory instruction (1:50), IgG antibody was stained as control. The tissues were blindly assigned to two professional pathologists for examination.

Total RNA was extracted from cell lines by utilizing TRIzol reagent (Invitrogen, USA). The first-strand cDNA was synthesized by using High-Capacity cDNA Reverse Transcription Kit (ABI, USA). RT-primers of RGS3 mRNAs were synthesized as follows: 5’- GCATCCTCTCTGCCCTTCTC -3’ (forward) and 5’- CTGGCTCGTGGAAGCTGTA -3’ (reverse) by Sangon Biotech Company (Shanghai, China). Real-time quantitative polymerase chain reaction (qRT-PCR) was performed according to TaqMan Gene Expression Assays protocol (ABI, USA).

### Prediction and validation of direct interaction between ROBO1 and microRNAs

By using online database miRDB (http://mirdb.org) and TargetScan, (http://www.targetscan.org), we suspected several molecular interactions with ROBO1, especially for post-transcriptional regulator. As analyzed, microRNA-218 (miR-218) showed a potential direct interaction with 3’ UTR of ROBO1 mRNA. Then, Dual-luciferase report assay was carried out to examine the exact interaction between miR-218 and ROBO1. A 202 bp sequence from the 3’-UTR of ROBO1 mRNA including the putative miR-218 binding site was selected as follow: 5’-tcaactttcagaagtgccacttaaggaagtttgatttttgtttttgtaatgcactgtttttaatctctctctctttttttttttttttttggttttaaaagcacaatcactaaactttatttgtaaaccattgtaactattaaccttttttgtcttattgaaaaaaaaaatgttgagaagcgtttttaacctgttttgttaa-3’. The corresponding mutant sequence were constructed by Sangon Biotech Company (Shanghai, China) as follow: 5’- tgatcatactgtactccgagtaatgcatgatagttatatctatatcttatggagtctatattaacacacacacatatatatatatatatagctataatatggagattgagttatcataaatagaatagcttagaatcaaattagcatatatctgtaaatcatatatatattctagtgtaccctatattagcagatatctaat -3’. Sequences above were cloned into pMIR-REPORT luciferase vectors (Promega, USA), containing Firefly luciferase, and pRL-TK vectors containg Renilla luciferase were used for control. Hep3B cells over-expressing miR-2186 or the negative control were co-transfected with vectors above, and luciferase activity were measured by Dual-Glo Luciferase assay system (Promega, USA) 48h post-transfection.

### Statistical analysis

Data from GEO and TCGA database was processed as mentioned above. Results derived from samples and cells were statistically analyzed by using SPSS 18.0. *P* values were calculated by paired t-test and Fisher’s exact text. *P* values < 0.05 were considered statistically significant.
